# Editorial: Exploring the role of epigenetic modifications in pulmonary vascular disease pathogenesis

**DOI:** 10.3389/fmed.2025.1618278

**Published:** 2025-05-16

**Authors:** Sasha Z. Prisco, Soban Umar, Francois Potus, Vineet Agrawal

**Affiliations:** ^1^Lillehei Heart Institute, Cardiovascular Division, Department of Medicine, University of Minnesota, Minneapolis, MN, United States; ^2^Division of Molecular Medicine, Department of Anesthesiology and Perioperative Medicine, David Geffen School of Medicine, University of California, Los Angeles, Los Angeles, CA, United States; ^3^Pulmonary Hypertension Research Group, Québec Heart and Lung Institute Research Centre, Québec City, QC, Canada; ^4^Department of Medicine, Laval University, Québec City, QC, Canada; ^5^Division of Cardiovascular Medicine, Department of Medicine, Vanderbilt University School of Medicine, Nashville, TN, United States; ^6^Department of Veterans Affairs, Tennessee Valley Healthcare System, Nashville, TN, United States

**Keywords:** epigenetics, pulmonary arterial hypertension, pulmonary vascular disease, pulmonary hypertension, right ventricle (RV), histone modification, noncoding RNA

Pulmonary arterial hypertension (PAH) is a progressive and life-threatening vasculopathy characterized by remodeling of the pulmonary vasculature, ultimately leading to right ventricular (RV) failure and death. Despite significant advancements in pharmacologic therapies over recent decades, current treatments remain insufficient to halt disease progression or significantly extend survival. Existing therapies predominantly target vasodilation, yet fail to address key pathogenic mechanisms, including dysregulated pulmonary vascular metabolism and mitochondrial dysfunction, excessive smooth muscle cell proliferation and apoptosis resistance, extracellular matrix remodeling and stiffening, and endothelial-to-mesenchymal transition.

**Figure 1:**
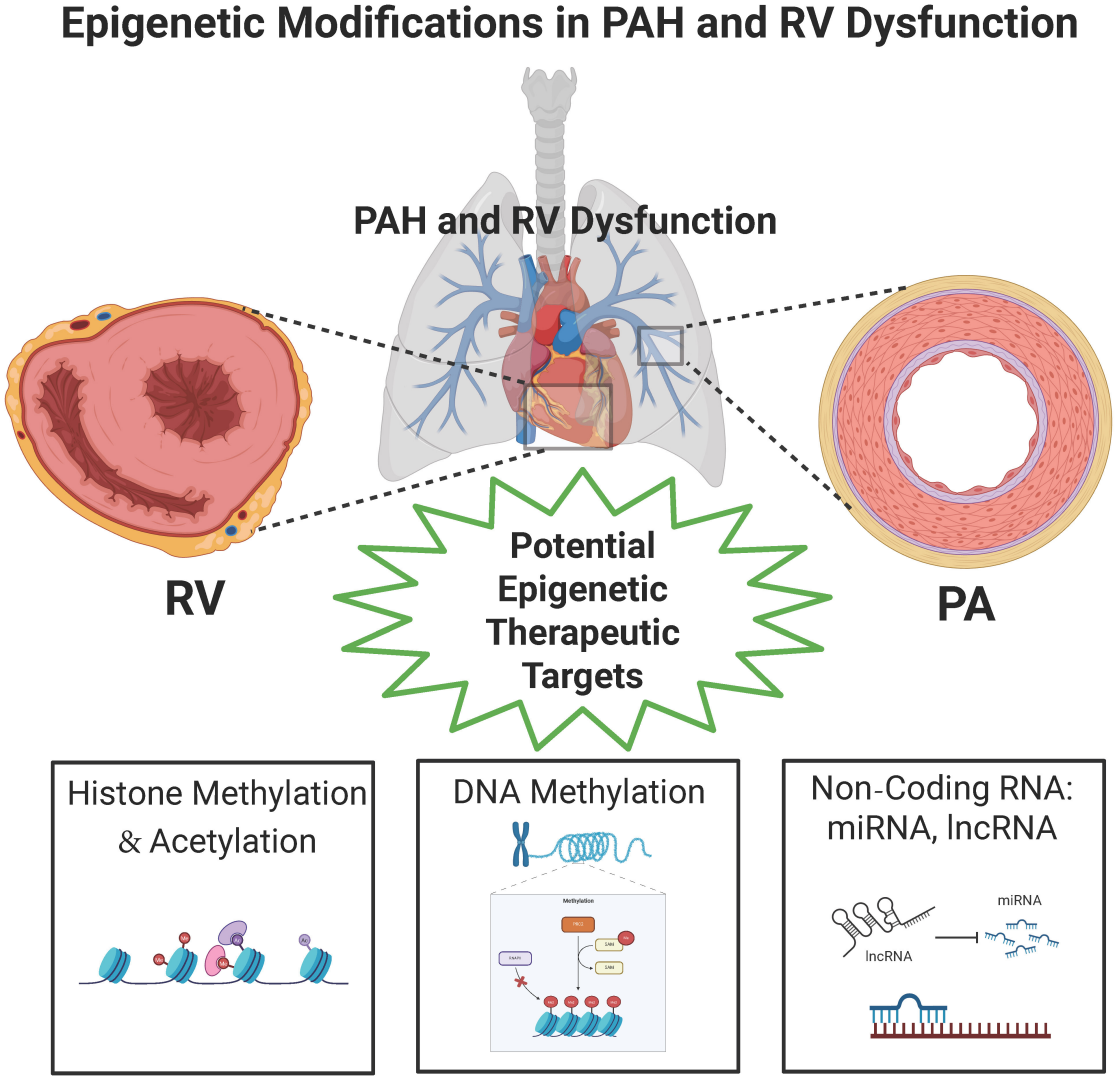
Potential epigenetic therapeutic targets in PAH and RV dysfunction. PAH, pulmonary arterial hypertension; lncRNA, long non-coding RNA; miRNA, microRNA; PA, pulmonary artery; RV, right ventricle. Created with Biorender.

Over the past decade, epigenetic modifications, defined as heritable or acquired changes that regulate gene expression without altering the underlying DNA sequence, have been increasingly recognized as key contributors to the pathophysiology and progression of cardiovascular diseases. Epigenetic mechanisms act as dynamic sensors, translating environmental cues such as diet, chemical or toxin exposure, medications, stress, and physical activity into gene regulatory changes. Advancements in high-throughput sequencing technologies have accelerated our understanding of how epigenetic dysregulation contributes to a range of chronic conditions. In PAH, accumulating evidence over the last 10 years has implicated multiple layers of epigenetic control in disease pathogenesis. These include aberrant DNA methylation (1); alterations in histone methylation and acetylation (2–7); and dysregulation of non-coding RNAs, including microRNAs (8–12) and long non-coding RNAs [(13–16); Figure 1]. As a result, epigenetics have emerged as a promising field in PAH research, offering new insights into disease mechanisms and novel therapeutic opportunities. Targeting epigenetic alterations holds significant potential as a precision medicine approach, paving the way for innovative, disease-modifying strategies in PAH.

Given the growing recognition of epigenetic dysregulation as a key driver of PAH, this special Research Topic, entitled “*Exploring the role of epigenetic modifications in pulmonary vascular disease pathogenesis*,” brings together timely contributions that not only synthesize current knowledge but also uncover emerging mechanisms linking epigenetic regulation to vascular remodeling in PAH. In this Research Topic, the review by Ejikeme and Safdar provides a comprehensive overview of the molecular pathways underlying vascular remodeling in PAH, with a particular focus on epigenetic regulators such as DNA methylation, histone methylation and acetylation, and non-coding RNAs. Their work highlights how these epigenetic mechanisms contribute to aberrant smooth muscle and endothelial cell phenotypes characteristic of the PAH vasculature. Importantly, they contextualize these epigenetic changes within broader disease-associated signaling networks, including mitochondrial dysfunction, hypoxia, estrogen signaling, and the BMP/TGF-β axis. Mitra et al. build on this foundation with a more focused review of the central role of hypoxia-inducible factors (HIFs) in modulating epigenetic programs. Their article discusses how hypoxic signaling regulates non-coding RNA expression and alters DNA and histone modifications. Notably, they also explore the reciprocal relationship, wherein epigenetic alterations can feedback to influence HIF stability and activity through post-translational modifications and chromatin remodeling. Finally, Hemnes et al. contribute original research that uncovers a novel link between metabolic reprogramming and epigenetic regulation in PAH. Their study demonstrates that altered lactate handling influences disease progression, supporting emerging evidence that lactylation, a recently described histone modification, may represent a key epigenetic mechanism coupling metabolism to RV dysfunction in PAH.

Beyond synthesizing the current literature on epigenetic modifications in PAH, a central aim of this Research Topic is to highlight critical gaps in our understanding of how environmental and acquired factors interact with genetic predisposition to shape the PAH phenotype through epigenetic mechanisms. Despite progress, major questions remain regarding the extent to which metabolic reprogramming, such as increased lactate production and subsequent histone lactylation, triggers transcriptional programs that drive maladaptive remodeling of the pulmonary vasculature and RV. Further investigation is also needed to elucidate the complex and multifaceted role of sex as a biological variable, particularly in modulating the penetrance and severity of genetic risk via epigenetic regulation. Other pressing gaps include a limited understanding of how acquired risk factors (e.g., obesity, diabetes) and environmental influences (e.g., gut microbiome composition) contribute to disease pathogenesis through epigenetic pathways. Moreover, the safety and therapeutic potential of both broad-spectrum and targeted pharmacologic strategies aimed at modifying epigenetic marks remain largely unexplored. Finally, it is unclear whether the epigenetic mechanisms identified in PAH extend to other forms of pulmonary hypertension, such as those associated with left heart disease or chronic lung disease. We hope this Research Topic serves as a catalyst for future mechanistic research aimed at closing these critical knowledge gaps and advancing the identification of novel therapeutic targets to prevent or mitigate the realization of heritable risk in pulmonary hypertension.
